# Cerebellar mutism syndrome: the importance of preoperative language assessment

**DOI:** 10.1007/s00381-022-05497-5

**Published:** 2022-03-25

**Authors:** S. Catelan, B. Santini, F. Sala, A. Feletti

**Affiliations:** Department of Neurosciences, Biomedicine and Movement Sciences – Neurosurgery Unit , University of Verona, Piazzale Stefani 1, 37126 Verona, Italy

**Keywords:** Cerebellar mutism syndrome, Preoperative neurocognitive assessment

## Abstract

Children undergoing surgical removal of tumors in the posterior cranial fossa can encounter a varied and complex constellation of neurological symptoms, called cerebellar mutism, defined as a disturbance in the planning and programming of motor language with preserved understanding, behavioral disorders such as inattention, visual-spatial disorganization, personality change, as well as ataxia and dysmetria. In the last years, several groups have been trying to establish risk factors or even predictive scores in order to be able at least in part to predict the appearance of speech disorders before surgery. We report on a child with pilocytic astrocytoma of the cerebellar vermis who had already been diagnosed with developmental linguistic delay two years earlier. This disorder initially worsened after surgery and later improved in the following 12 months. The aim of this paper is to emphasize the importance of preoperative neuropsychological evaluation. The present case, along with those reported in the literature, suggests that the risk of long-term cerebellar mutism is higher in children with preoperative speech disorders. In these patients a thorough assessment of cognitive and linguistic functions is therefore necessary to better evaluate the risk of cerebellar mutism after surgery.

## Introduction

Cerebellar mutism syndrome (CMS) is a well-known condition that may occur in patients after posterior fossa surgery. CMS usually appears one or two days after surgery. Symptoms include loss of speech, loss of muscle tone, oropharyngeal dysfunction with dysphagia, loss of balance, mood swings, and changes in personality. Many of these symptoms improve over time [1]. It is usually considered a possible complication of surgery for posterior fossa tumors, being children at higher risk [2].

Interestingly, some children with posterior fossa tumors may present with a preoperative language impairment. In this paper we report on a case of preoperative language impairment in a child with a cerebellar tumor, and we review the literature about this sometimes subtle preoperative symptom, with the aim to emphasize the importance of preoperative language and cognitive assessment.

## Case report

A 3 years and 3 months old, right-handed male child underwent neuropsychological assessment due to language delay with the following tests: child audiology evaluation, MacArthur-Bates Communicative Development Inventory-CDI survey, socio-conversational skills, cognitive, and logopedic rating.

The examination showed a delayed lexical development, decontextualized understanding, but not decontextualized production. He was an “idle conversationalist” with a low level of both assertive and responsive behavior. A Wechsler Preschool and Primary Scale of Intelligence test was submitted to the child, but he was not compliant and preferred solitary play. Moreover, in the speech therapy evaluation he struggled to accept to be tested, therefore not all tests required by the screening were performed. The verbal comprehension skills were below the average for lexical aspects, and it was therefore not possible complete the morpho-syntactic understanding tests. In particular, his lexical development was consistent with an age of 30 months regarding names and 23 months regarding the predicates. Even language production was below the average for his age.

Overall, he had a developmental delay with adjustment difficulties. Language delay was related to both production and verbal comprehension. Psychoeducational therapy was proposed.

Two months later he presented with projectile vomiting and headache, without fever. These symptoms were treated as influenza. After another two months he became unstable in walking, so his parents took him to the hospital.

At admission, physical examination of the child was normal, but the neurological examination revealed unstable walking and delay of speech, compared with age norms. Blood tests revealed no abnormalities, apart from his hormonal profile, which showed an elevated IGF-1 39.33 nmol/L. The chest X-ray, ECG, and transthoracic echocardiography were unremarkable.

**Fig. 1:**
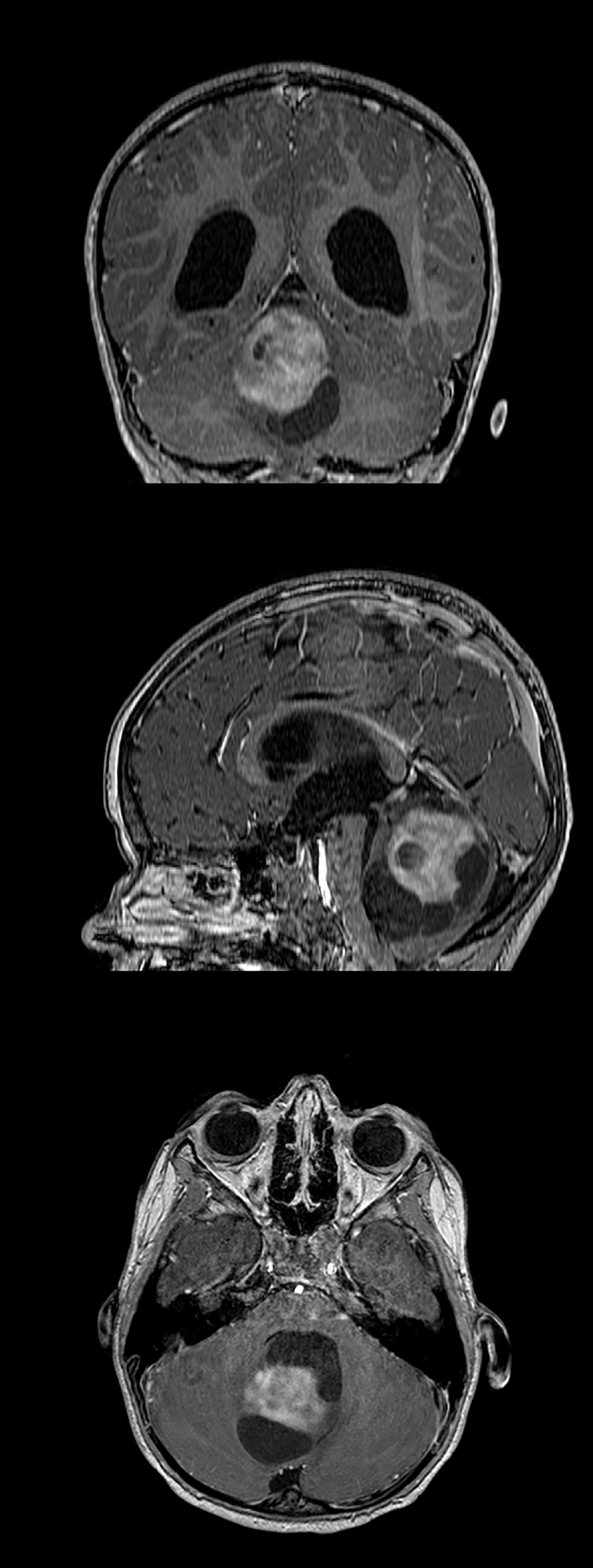
T1-weighted, gadolinium-enhanced coronal, sagittal, and axial cerebral MRI showing a large vermian mass with well-defined margins of approximately 57 × 50 × 44 mm. The mixed structure with a solid, gadolinium-enhancing nodule and multi-lobed cystic component was consistent with the diagnosis of pilocytic astrocytoma. Triventricular hydrocephalus and initial descent of the cerebellar tonsils are also evident

A brain MRI showed a large vermian mass with well-defined margins of approximately 57 × 50 × 44 mm. It showed a mixed structure with a solid component and an inhomogeneous appearance and intense gadolinium uptake and a further multi-lobed cystic component, consistent with the diagnosis of pilocytic astrocytoma (PA) (Fig. 1). Triventricular hydrocephalus and initial descent of the cerebellar tonsils were also detected.

A median sub-occipital craniotomy with removal of the lesion was performed, and an external ventricular drainage (EVD) was placed. Histopathological analysis confirmed a PA (WHO I).

The speech disorder severely worsened immediately after surgery. The child was only able to pronounce “mom” and “no.” From the fifth day after surgery, there was a gradual progressive improvement, with a clearer and faster speech, although at a lower level than preoperatively. He underwent speech therapy sessions, and his speech was significantly improved at the 15-month follow-up. He was able to climb the stairs, walk autonomously, and had improved balance after rehabilitation therapy. The latest brain MRI control ruled out any tumor residue or relapse.

## Discussion

In the last years, several studies clarified risk factors for development of postoperative CMS in children after posterior fossa surgery [3–6]. However, it is nowadays evident that some children affected by posterior fossa tumors experience speech disorders even before surgery. Although only a few papers [7–10] have reported preoperative speech disorders in children, their prevalence is probably higher. The underestimation of this problem is possibly due to the fact that tailored preoperative neuropsychological tests are not routinely performed.

The most relevant and comprehensive set of tests to evaluate preoperative speech disorders is summarized in Table 1. As reported by Di Rocco et al., the presence of speech disorders in patients with lesions in the posterior cranial fossa is a predictive factor for the development of postoperative CMS [7]. In their study, children undergoing surgery for posterior fossa lesions were divided into two subgroups depending on whether or not they had preoperative speech disorders. In the group in which speech disorders were already present, CM occurred in 99% of cases, compared to 65% in the group that did not have preoperative speech alterations.

**Table 1:** Most commonly used neuropsychological tests in children. The tests, based on the patient’s age, are used to assess the degree of cognition, praxia, and language (based on Di Rocco et al. [7])

	**Children younger than 5 years**	**Children older than 5 years**
Intellectual profile	Griffiths Mental Developmental Scale Revised (GMDS-R). Wechsler Preschool and Primary Scale of Intelligence and Leiter-R for re-test;	
Cognitive development	Leiter-R;	Wechsler Intelligence Scale for Children Revised or third revision (WISC-R or WISC-III); Leiter-R;
Language skills	First Language Test (FLT) and Mean Length of Utterance (MLU);	• Language dynamics, tested by MLU; • Naming and comprehension, tested by FLT, Language Assessment Test (LAT), and the Boston Naming Test; • Syntactic knowledge, tested by Peabody Picture Vocabulary; • Phonological and semantic retrieval, tested by Verbal fluency; • Phonological generation, tested by LAT; • Speech apraxia, tested by orofacial articulatory planning
Visual attention	Teddy bear and attention sustained, tested by Leiter-R	Bell’s cancellation, visual selective attention, tested by Leiter-R;
Visual constructional/memory	Rey Copy Recall;	VMI, Beery/Rey complex figure, Rey copy/Block design;
Working memory	Digit span, tested by GMDS-R;	Manual Motor Imitation, testing procedural memory, Learning List, Test Of Memory And Learning, testing verbal and visual memory, and digit span for working memory;
Executive functions		Tower of London and imaging (sequential order; picture arrangement);
Visual constructional	Visual Motor Integration (VMI) and Rey Copy;	
Measures of behavioral/affective regulation	Achenbach’s Child Behavior Checklist (CBCL; 11⁄2–5 years);	CBCL;
Motor skills	Posting coins, in the context of ABC movement;	ABC movement

Another study, by Turkel et al., showed that among 22 children undergoing surgery for posterior fossa tumors, some already presented with mutism (21%), and some with a history of mood or behavior symptoms as apathy, depression, dysphoria, irritability, and inattention. The authors also noted that children with CMS symptoms before surgery had more pronounced CMS after surgery and more persistent postoperative cognitive alterations [10]. Similarly, in the study published by Bianchi et al. the analysis of the long-term outcome showed the tendency to persisting language disturbances at 2 years in children presenting with language disturbances preoperatively [8]. Besides these series, Chen et al. reported a case of preoperative speech disorder in a child with posterior fossa pilocytic astrocytoma [9]. Interestingly, not only neoplastic masses can lead to this syndrome, as described by Baillieux et al., who reported on a case of transient cerebellar mutism associated with cognitive-emotional and behavioral symptoms after rupture of a cerebellar vermian AVM in a 12-year-old boy. In their case report they hypothesized structural damage to the dentate-thalamocortical pathway [11].

The etiopathology to explain preoperative language impairment is probably similar to the mechanisms proposed to justify postoperative CMS. Our patient was affected by a pilocytic astrocytoma, which had likely been growing for some time before causing neurological symptoms leading to diagnosis. During this period, the tumor might have played a causative role in the preoperative speech impairment. In this perspective, the eventual appearance of a speech disorder should not be underestimated, as it might be a sign of a tumor in the cerebellum. Various studies have demonstrated that the cerebellum has important functions regarding working memory, spatial and language tasks, and in addition to a "motor" projection, there are projections toward the posterior parietal cortex and the prefrontal area that contribute to visuospatial function and cognitive aspects [12–14]. It has been shown that patients with tumors located higher in the fourth ventricle are statistically more likely to develop mutism and other symptoms related to posterior fossa syndrome, probably because there is more preoperative stress on the proximal dentatothalamocortical (DTC) tract [15]. The DTC connects the supplementary motor cortex and the dentate nuclei through the ventrolateral nucleus of the thalamus. As a damage of any of these structures can lead to mutism, it is likely that the interruption of the DTC can have a role in the etiopathogenesis of cerebellar mutism [16]. Interestingly, the study by Morris showed that in patients affected by CMS more than 3 structures are involved among the pons, dentate nucleus (DN), superior cerebellar peduncles and midbrain, bilaterally. They concluded that multiple and bilateral damage to the tract is necessary to have symptoms [15]. The review of the literature reveals that 41 of 58 children with preoperative speech disorder had midline, vermis, or IV ventricular lesions; 7 children had midline lesions with lateral extension; and 10 had lesions in the cerebellar hemisphere. In the Bianchi’s series, including 20 children presenting with speech disorders in the preoperative period, 7 had invasion of the brainstem and 17 had DN damage, confirming the importance of this structures in the etiopathogenesis of CMS [8].

This evidence underlines the importance of a thorough evaluation of patients with a posterior fossa mass that is scheduled for surgical removal, not only because of the postoperative risk of a speech disorder, but also because of a possible cognitive and neurological impairment. Obviously, in many cases it is very difficult or even not possible for the child to complete the proper neuropsychological assessments. However, clinicians should always be aware of the possibility of a preoperative language impairment and consequently try to have assessed in as much detail as possible the language status and development of children at risk of postoperative CMS. This would also allow for a more informed consenting process with parents.

## Conclusion

CMS is a complex syndrome most typically encountered as a consequence in children operated on for posterior fossa lesions. The presented case, along with those reported in the literature, highlights the importance of preoperative language evaluation of children who are candidates for neurosurgical removal of posterior cranial fossa tumors, because even a mild preoperative language disturbance can significantly get worse in the postoperative period. For this reason, a thorough preoperative neurocognitive evaluation is of the utmost importance and should be routinely performed in these cases, to identify patients at higher risk.

## Abbreviations

CMS: Cerebellar mutism syndrome
PA: Pilocytic astrocytoma
EVD: External ventricular drainage
WHO: World Health Organization,
MRI: Magnetic resonance imaging
CM: Cerebellar mutism
DN: Dentate nucleus
DTC: Dentatothalamocortical
AVM: Arteriovenous malformation
